# Functionalization of Nanomaterials for Skin Cancer Theranostics

**DOI:** 10.3389/fbioe.2022.887548

**Published:** 2022-04-26

**Authors:** Chao Zhang, Xinlin Zhu, Shuming Hou, Weihua Pan, Wanqing Liao

**Affiliations:** ^1^ Department of Dermatology, Changzheng Hospital, Naval Medical University, Shanghai, China; ^2^ Orthopaedic Oncology Center, Department of Orthopedics, Changzheng Hospital, Naval Medical University, Shanghai, China

**Keywords:** functionalization, nanomaterials, skin cancer, theranostics, advances

## Abstract

Skin cancer has drawn attention for the increasing incident rates and high morbidity worldwide. Timely diagnosis and efficient treatment are of paramount importance for prompt and effective therapy. Thus, the development of novel skin cancer diagnosis and treatment strategies is of great significance for both fundamental research and clinical practice. Recently, the emerging field of nanotechnology has profoundly impact on early diagnosis and better treatment planning of skin cancer. In this review, we will discuss the current encouraging advances in functional nanomaterials for skin cancer theranostics. Challenges in the field and safety concerns of nanomaterials will also be discussed.

## 1 Introduction

Skin is a physical barrier made up of cells and intercellular matrix that is robust and long-lasting (Brogden et al., 2012). In humans, skin malignancies display a recurring malignant response in a significant number of cases, with over one million cases reported, with white individuals accounting for the majority of cases (D’Orazio et al., 2013). Ultraviolet (UV) radiation, a key contributing factor in the development of skin photoaging, causes uncontrolled cell growth and the death of keratinocytes (Lopes et al., 2021).

Skin cancers can be categorized into melanoma skin cancers (MSC) and non-melanoma skin cancers (NMSC) (Abi Karam et al., 2021). NMSC are then classified into basal cell carcinoma (BCC), squamous cell carcinoma (SCC), and Merkel cell carcinoma (MCC) (Armstrong and Kricker, 2001; Kaur and Kesharwani, 2021; Thai et al., 2021). NMSC are the most commonly occurring cancers worldwide, in which BCC account for 75% and SCC for 20%, respectively (Esteva et al., 2017). Meanwhile, MSC are responsible for a high amount of fatalities, putting a significant strain on medical services (Carlino et al., 2021).

According to the American Academy of Dermatology clinical practice guideline, diagnostic skin biopsy maintains the first line to identify MSC (Swetter et al., 2019). NMSC are generally diagnosed clinically, with histological confirmation after excision (Newlands et al., 2016). Nevertheless, there is the paucity of clinical practice to specifically and accurately diagnose tumor metastasis (Dinnes et al., 2018). Conventional treatment of the primary lesions involves surgical excision, cryotherapy, radiation therapy (RT), and topical agents (Swetter et al., 2019). Once metastasis occurs, chemotherapy, adjuvant immunotherapy, and targeted therapy are suggested (Majem et al., 2021). However, the potential accompanied disadvantages of chemotherapies, including normal cell damage, relatively low bioavailability, and tumor drug resistance, cannot be ignored (Kwon et al., 2021; Taeb et al., 2021; Tarik Alhamdany et al., 2021). As a result of these restrictions, skin cancer theranostics are unsatisfactory.

In recent decades, nanomaterials have given rise to a new discipline, which has gotten a lot of interest in the field of cancer theranostics (Song et al., 2010, 2016; Liu et al., 2018). Certain nanomaterials could concentrate in tumor primary site, lymph node metastases, and distant metastasis, which provides the ability for targeted imaging and efficient anti-cancer effect (Chen and Cai, 2014; Zhang P. et al., 2021; Gracia et al., 2021). In comparison to conventional nanomedicine, functionalized nanoparticles have several benefits, including increased therapeutic effectiveness and delivery, increased drug solubility, improved pharmacokinetic profile, and prolonged blood circulation time. Chemical and biofunctionalization are currently two strategies for the modification of nanoparticles. In addition, these advanced nanotechnologies assist the stabilization of anti-cancer drugs, which improve the bioavailability and controlled release (Chen and Stephen Inbaraj, 2019; Jokioja et al., 2021; Rashwan et al., 2021; Shen et al., 2022).

## 2 DILEMMA OF SKIN CANCER THERANOSTICS

### 2.1 Melanoma Skin Cancers

MSCare caused by abnormal melanogenic cells called melanocytes, which proliferate excessively and spread invasively (Molodtsov et al., 2021). Although most melanomas are pigmented, around 5–10% of cutaneous melanomas are amelanotic, which may cause delayed detection and worse prognosis (Chen and Sebaratnam, 2021; Chuchvara et al., 2021). From dermoscopy through reflectance confocal microscopy (RCM) to histology, this condition emphasizes difficulties in the diagnosis of amelanotic melanoma (Li et al., 2021; Rasmussen et al., 2021; Williams et al., 2021).

MSChave a predisposition for migrating to the brain and central nervous system, causing considerable morbidity and treatment resistance (Johnson and Young, 1996). Patients with active brain metastases were excluded from most of the clinical studies, despite the fact that anti-programmed cell death protein 1 (anti-PD1) and anti-cytotoxic T-lymphocyte antigen 4 (anti-CTLA4) immunotherapies and mitogen-activated protein kinase-targeted (MAPK-targeted) treatments have been extensively employed in the treatment of systemic metastases from melanoma (Eroglu et al., 2019).

The major molecular signaling pathways in MSC include MAPK signaling pathway (Falchook et al., 2012; Cancer Genome Atlas Network, 2015), phosphatase and tensin homolog (PTEN)/phosphoinositol-3-kinase (PI3K) signaling pathway (Damsky et al., 2015), Rac family small GTPase 1 (RAC1) signaling pathway (Krauthammer et al., 2012), and cyclin dependent kinase inhibitor 2A (CDKN2A) (Van Raamsdonk et al., 2009; Kwong et al., 2012; Horn et al., 2013; Mittal and Roberts, 2020). Accordingly, mitogen-activated protein kinase-kinase (MEK) inhibitors (B-Raf proto-oncogene, serine/threonine kinase) BRAF inhibitors, and immunomodulation are recommended for MSC that has progressed to stage IV (Hodi et al., 2010; Villanueva et al., 2010; Chapman et al., 2011; Larkin et al., 2014). Targeted therapy has a high response rate and can improve survival in most MSC cases, patients nearly always relapse and die from the illness (Ahronian et al., 2015; Samson et al., 2019; Foth and McMahon, 2021).

According to published studies, several drug-resistance mechanisms of target therapy have been found and may be categorized as follows: 1) reactivation of MAPK signaling pathways (Poulikakos et al., 2011; Shi et al., 2012, 2014; Johnson et al., 2018), 2) upregulation of p21-activated kinase (PAK) signaling pathway and growth factor receptors (Johannessen et al., 2013; Lu et al., 2017), and 3) low melanocyte inducing transcription factor (MITF) and dedifferentiated cell states (Hoek et al., 2006; Smith et al., 2016). To overcome the therapeutic resistance, inhibition of pathways mentioned above is attempted in clinical trials (Nathanson et al., 2013; Sullivan and Flaherty, 2015; Romano et al., 2018; Sullivan et al., 2018). Nevertheless, inhibition of cyclin dependent kinase4/6 (CDK4/6) and MEK is further limited by adverse effects that need a lower dose, leading to diminished effectiveness and susceptibility to resistance development (Kwong et al., 2012; Guo et al., 2021).

Immunotherapy has revolutionized the treatment of numerous malignancies, probably the most advanced melanoma (Sheng et al., 2021). In Eggermont et al.‘s study, 200 mg of pembrolizumab given every 3 weeks for up to a year as adjuvant therapy for high-risk stage III melanoma resulted in considerably longer recurrence-free survival than placebo, with no new adverse effects (Eggermont et al., 2018). Pembrolizumab adjuvant therapy produced a persistent and clinically substantial improvement in recurrence-free survival in resected high-risk stage III melanoma at a 3-years median follow-up (Eggermont et al., 2020). However, initial and acquired resistance to the therapy inevitably shorten survival and greatly compromised the quality of life in these patients. The underlying mechanisms associated with drug resistance include immune surveillance escape, interferon signaling deficiency, and MITF^low^/dedifferentiated phenotype (Sosman et al., 2012; Sade-Feldman et al., 2017; Liu et al., 2019).

Because of the restrictive method including numerous medications, a novel technique for treating melanoma is desperately required (Bowman et al., 2021; Millán-Esteban et al., 2021; Teixido et al., 2021).

### 2.2 Non-Melanoma Skin Cancers

The majority of NMSC may be efficiently treated with surgery; however, less than 10% of cases are progressed and may need further therapy (Zaar et al., 2016). From an epidemiological viewpoint, SCC and BCC are known as keratinocyte carcinomas, owing to keratinocyte genesis. BCC seldom metastasizes, but commonly features with histological invasion of adjacent tissues, thereby causing significant morbidity (Cameron et al., 2019). The high mortality rate of SCC is mainly due to the complications of metastasis (Apalla et al., 2017). MCC, a rare cancer of the skin, is an aggressive neuroendocrine skin cancer (Kim et al., 2021). Despite the therapeutic efficacy, the increasing incidence of NMSC entails a large health and economic burden worldwide.

#### 2.2.1 Basal Cell Carcinoma

The conventional surgical operation remains the first-line therapy of BCC, which is based on multidisciplinary collaboration. Knowing more about the molecular mechanisms contributing to BCC progression can help us develop more effective therapeutic regimens. Among the important molecular pathways in BCC development is the Hedgehog (HH) signaling pathway (Wong and Reiter, 2008). Based on the mechanisms activated along the HH pathway, several targeted therapies have been developed to apply for advanced BCC (Dessinioti et al., 2014). In the clinical trial of HH inhibitors, adverse events occur in roughly 30% of patients, which include fatigue, weight loss, dysgeusia, and so on (Sekulic et al., 2012; Sharpe et al., 2015).

#### 2.2.2 Squamous Cell Carcinoma

The invasive form of SCC is the second most frequent kind of NMSC, accounting for 20% of all cutaneous malignancies (Rogers et al., 2010). Before metastasizing to distant locations, invasive SCC commonly spread to lymph nodes located in the vicinity. When distant metastasis occurs, patients usually suffer from a poor prognosis. As a result, it is critical to maintain SCC’s generally high odds of cure by carefully evaluating and managing all instances early on, and not to underestimate the tumor’s potential for aggressiveness.

When the tumor arises *de novo* or the early keratosis phase is lacking, SCC can present as an asymptomatic small plaque or nodule that enlarges over time. Tumor extension or infiltration may extend beyond the visible borders of the lesion, which may create difficulties for diagnosis.

The progression of SCC follows a multistage malignant transformation paradigm. Further mutational and cellular processes will result in invasive growth and, less often, metastasis. The most prevalent genetic abnormalities detected in SCC are mutations in the tumor suppressor gene p53 (Yan et al., 2021). A significant proportion of p53 mutations is localized opposite pyrimidine dimer sites (C–C) and likely derives from UV exposure (Boukamp, 2005). Aberrant activation of epidermal growth factor receptor (EGFR) and Fyn leads to downregulation of p53 mRNA and protein levels *via* a c-Jun dependent process, indicating another method for modulating p53 activity. (Zhao et al., 2009, 53).

Surgery is the gold standard treatment for SCC, although other treatments include laser dissection, intra-lesion medication injection, and electrodissection. In patients who were deemed inappropriate for surgery due to comorbidities, original tumor location, the danger of local infiltration, or quality of curative margins, other options were examined. External beam RT and brachytherapy are two of them.

The poor results of conventional chemotherapy in patients with advanced SCC, as well as the findings of original studies revealing a high number of genetic abnormalities and neo-antigen load, led to the development of clinical trials with immune checkpoint inhibitors (primarily PD-1) in patients who were not candidates for other treatments. Programmed cell death ligand 1 (PD-L1) levels did not correlate with clinical response to anti-PD1 mAbs in a manner comparable to melanoma). As a result, cemiplimab and pembrolizumab, immune checkpoint inhibitors, have been licensed in the United States for the treatment of locally progressed or metastatic SCC (Migden et al., 2018; Hernandez-Guerrero et al., 2019; Migden et al., 2020). Systemic chemotherapy is currently not licensed for SSC because of the low response rates and the high expense of major side effects, particularly in a vulnerable patient group.

#### 2.2.3 Merkel Cell Carcinoma

Despite its rarity, MCC are becoming more common, owing to advances in detection as well as the worldwide population’s aging. The incidence of MCC rose from 0.5/100,000 people in 2000 to 0.7/100,000 people in 2013, of which the data are obtained from the Surveillance, Epidemiology, and End Results (SEER) database (Paulson et al., 2018).

MCC is difficult to diagnose clinically, requiring nearly exclusively on histological investigation. Immunohistochemistry seems to be effective in the differential diagnosis of MCC. Functional imaging is now regarded as the gold-standard approach for the clinical evaluation of MCC at diagnosis and follow-up because of the superior sensitivity of ^18^F-fluoro-2-deoxyglucose-positron emission tomography/computed tomography (^18^FDG-PET/CT) imaging compared to CT or magnetic resonance imaging (MRI) (Concannon et al., 2010).

The stage of the illness and the kind of lymph nodes affected to determine how MCC patients are treated. Sentinel lymph node biopsy (SLNB) is usually recommended in people who have no signs of lymph node involvement; in those who have lymphoma, a blood test may be necessary. In the first-line context, chemotherapeutic therapies such as platinum-based combos, etoposide, topotecan, taxanes, and anthracyclines were frequently utilized (Tai et al., 2000; Becker et al., 2017; Garcia-Carbonero et al., 2019). Chemotherapy’s immunosuppressive impact is now thought to be a plausible mechanism for the early development of resistance after cytotoxic treatment in the setting of a highly immunogenic malignancy (Pommier et al., 2004).

Understanding the pathophysiology of BCC, SCC, and MCC has enabled the development of innovative therapeutics, which have had a significant influence on patient survival and quality of life. The immune system plays an important role in SCC pathogenesis, and preclinical models have revealed important details regarding immune cell changes that govern skin cancer biology. Research on NMSC microenvironment abnormalities implies that malignant cells and those in charge of the innate or adaptive immune systems are in a constant state of interaction. The discovery of these events has gradually altered the landscape of metastatic SCC therapy, opening up new possibilities that are also being investigated in MCC and BCC. In this context, new approaches are being investigated in order to overcome current constraints, with the goal of tailoring therapy in response to the cancer cells’ ongoing phenotypic and antigenic changes.

## 3 FUNCTIONALIZED NANOMATERIALS FOR SKIN CANCER THERANOSTICS

Anticancer treatments are thought to fail owing to the negative effects of most anticancer medications, low drug concentrations at the tumor site, and the development of drug resistance. Because nanocarriers have the ability to selectively target afflicted organs and cells while sparing normal tissues, the application of functionalization methods, such as the production of nanocarriers for medication delivery or imaging agents, has earned a lot of interest (Marianecci et al., 2014). A number of functionalization techniques have been investigated to modify and functionalize the surface of nanoparticles for cancer theranostics applications (Ulbrich et al., 2016; Tomitaka et al., 2019). Surface modification of nanocarriers may possibly enhance the biological features of nanocarriers, building on the benefits of traditional nano-drug delivery methods (Frickenstein et al., 2021). Traditional nanoparticles are normally quickly opsonized and removed from the bloodstream by reticuloendothelial system (RES) macrophages, which are mostly found in the liver and spleen after intravenous administration (Neves et al., 2016). In this context, the functionalization of surfaces could enable nanocarriers to escape immune surveillance and break through the biological barriers. Chemical functionalization and biofunctionalization are the two main approaches of surface modification. Chemical functionalization has been achieved using a variety of chemical methods, including amide coupling (Giust et al., 2015), click reactions (Ulrich et al., 2021), thiol coupling (Fu et al., 2021), PEGylation (Park et al., 2021), etc. At present, bio-functionalization is confirmed to enhance the blood circulating duration, distribution of the drug, cellular uptake, and regulate immune response and intracellular trafficking (Tang et al., 2021).

### 3.1 Functionalized Liposomes

Liposomes are spherical vesicles with phospholipid bilayer membranes that are considered non-toxic and biodegradable carriers for the encapsulation and targeted administration of a variety of hydrophobic and hydrophilic medicinal substances (Samad et al., 2007; Chatzikleanthous et al., 2021). Their substantial utility as a replacement in delivering the therapeutic moiety to the targeted location has been utilized to broaden the therapeutic profile of anti-cancer medications while reducing the occurrence of adverse events (Jash et al., 2021). Liposomes are employed in a variety of applications, including biological imaging, fluorescent probes, and more (Erten et al., 2010).

In passive targeting, nanocarriers use the enhanced permeability and retention effect to accumulate within the tumor cells (Bazak et al., 2014). The US Food and Drug Administration (FDA) has authorized many passively targeted liposomal medicines for cancer therapy, which include daunorubicin, doxorubicin, paclitaxel and vincristine (Amreddy et al., 2018). Many studies have looked at the extent and technique of passively targeting liposomal-based medicinal substances for melanoma therapy. Despite the fact that liposomes are potential carriers for the delivery of medicinal substances, the development of liposomal drug delivery systems is currently hampered by a number of limitations. The rapid clearance of liposomes by the RES remains a major challenge. Liposomal absorption by the RES may be prevented by conjugating poly (ethylene glycol) (PEG) to the liposomal membrane. PEGylation, on the other hand, may result in the creation of anti-PEG IgM, causing the liposomes to lose their long-circulating properties and speeding up blood clearance. Immune responses or immunogenicity may also be elicited by PEGylated liposomes. For the purpose of clearance prevention, Fu et al. created a PEGylated PTX-loaded liposome (Fu et al., 2015). To overcome drug resistance and resistance-related metastases in melanoma, mitochondrial targeting topotecan-loaded liposomes have been produced (Yu et al., 2012). Liposome has also been utilized to deliver curcumin in tumors resistant to conventional therapy (Tomeh et al., 2019). Karewicz et al. found that chitosan-coated curcumin-loaded liposome exhibited more efficient anti-cancer effects than that of free curcumin (Karewicz et al., 2013). To address the constraints of photodynamic treatment (PDT), the nitrosyl ruthenium complex [Ru(NH.NHq) (tpy)NO]3+ (RuNO) was coencapsulated with ZnPc in ultradeformable liposomes (UDLs), which demonstrated better flexibility and skin penetration than traditional liposomes (de Lima et al., 2017).

**FIGURE 1:**
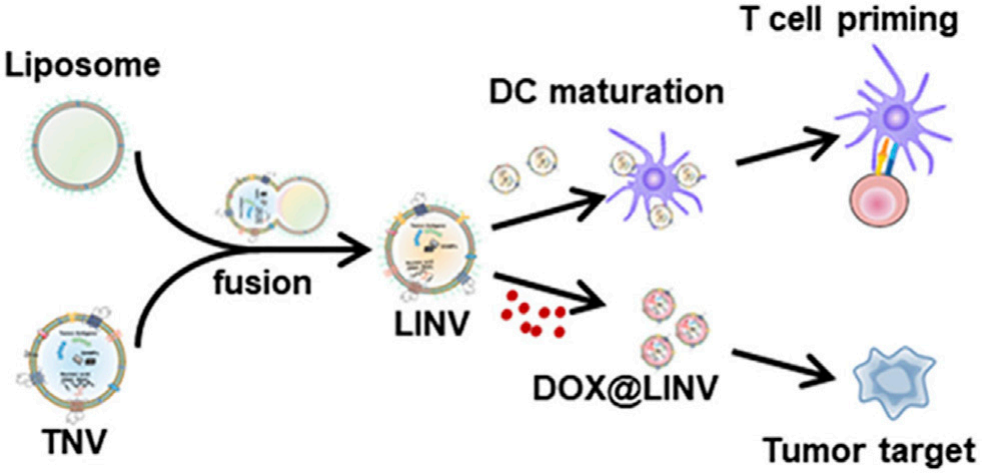
Illustration of the doxorubicin (DOX)-loaded biomimetic hybrid nanovesicles (DOX@LINV) *via* fusing artificial liposomes (LIPs) with tumor-derived nanovesicles (TNVs) for combinational immunochemotherapy. DOX@LINV with a homologous targeting ability could deliver DOX to tumor tissue and elicit an effective immunogenic cell death response to improve the immunogenicity of a tumor. Meanwhile, the preserved tumor antigens and endogenous danger signals in DOX@LINV activated dendritic cells and induced a subsequent antigen-specific T cell immune response (Hu et al., 2021).

Active targeting relies on the interaction of overexpressed receptors in tumor cells with ligands on surface-modified nanocarriers, such as antibodies, peptides, nucleic acids, and small molecules (Bi et al., 2016; Hu et al., 2021) (Figure 1). CD20+ melanoma stem cells are important for melanoma metastasis and initiation. As a result, targeted eradication of CD20+ melanoma stem cells is a viable strategy for melanoma eradication. Zeng et al. used a single-step nanoprecipitation approach to create salinomycin-loaded lipid-polymer nanoparticles with anti-CD20 aptamers (CD20-SA-NPs). Results indicated that CD20-SA-NPs (salinomycin 5 mg kg^−1^ d^−1^, iv, for 60 days) showed greater effectiveness in inhibiting melanoma development in mice carrying xenografts compared to SA-NPs and salinomycin (Zeng et al., 2018).

### 3.2 Functionalized Metal Nanoparticles

Functionalized metal nanoparticles for skin cancer theranostics are widely investigated, of which gold and silver are explored in-depth (Lin et al., 2014; Mukherjee et al., 2016; Gaddam et al., 2017).

In skin cancer treatment, gold nanoparticles (Au NPs) were employed for targeted medication administration, tumor progression monitoring, and vaccination (Sau et al., 2014; Mukherjee et al., 2016; Kotcherlakota et al., 2019; Meka et al., 2019). (Brown et al., 2010; Yeh et al., 2012; Gao et al., 2021). Au NPs are now being studied for medicinal purposes due to their potential to increase anticancer activity while reducing undesirable side effects (Kodiha et al., 2015; Katoozi et al., 2021; Tomşa et al., 2021).

Additionally, numerous studies have also explored the application of silver nanoparticles (Ag NPs) for skin-cancer theranostics (Lin et al., 2014; Mukherjee et al., 2014). Lin et al. found that increasing autophagy using Ag NPs helped cells survive, whereas suppressing autophagy with ATG5 siRNA enhanced cancer cell death (Lin et al., 2014). Due to the appearance of hazardous after-effects and the high cost of processing techniques employed in the manufacturing of silver nanoparticles (Ag NPs), the focus has switched to the adaption of green alternatives as a means of overcoming the obstacles posed by them. The extra benefit of imparting biocompatible action while simultaneously lowering the costs associated with the manufacturing of such nanocarriers makes this a viable option for treating uncontrolled skin cancers. Horse chestnut leaves, according to Küp et al., have reduction potential as well as the ability to act as a capping agent in the production of well-defined nanoscale silver particles (Küp et al., 2020).

Because of its effectiveness as a cancer treatment technique, PDT is becoming more popular. Organic photosensitizers used in PDT have a number of drawbacks, including high toxicity, non-selectivity for tumors, and low light absorption. Low light penetration into tumor areas due to low absorption wavelength and long-term skin photosensitivity. As a result, non-toxic inorganic photosensitizers such as noble metal nanoparticles are receiving more attention these days. Nanomaterials are replacing organic dyes since they have photostability and non-toxicity. Among the metal nanoparticles, noble metals, especially gold and silver are attractive because of their size and shape-dependent unique optoelectronic properties. The noble metal is coated with inorganic/organic compounds, making the nanoparticles biocompatible and less poisonous. Furthermore, because of their distinct architectures, Ag- and Au-based inorganic/organic complex nanoparticles may provide a new potential (Dhanalekshmi et al., 2020). Meanwhile, the coating of inorganic/organic complex nanoparticles shields and stabilizes noble metals against chemical corrosion while also increasing reactive oxygen species generation.

**FIGURE 2:**
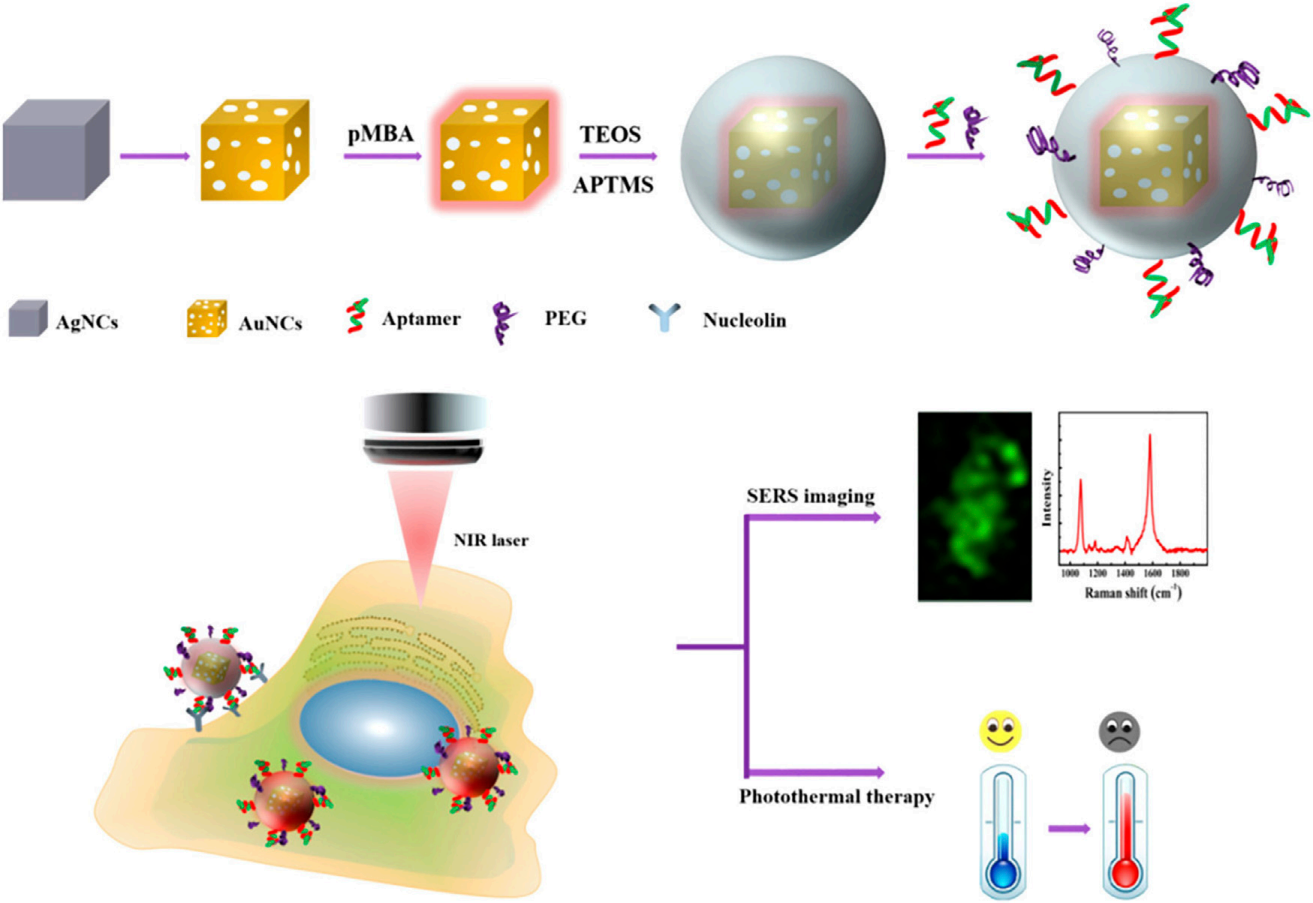
Schematic diagram of cellular SERS imaging and photothermal therapy (Wen et al., 2019)

Biopsy and radiography are not sensitive enough to identify melanoma in its early stages. In recent years, several attempts have been made to develop effective theranostics modalities that combine diagnostic and therapeutic roles to enhance cancer treatment (Andreiuk et al., 2022). Surface-enhanced Raman spectroscopy (SERS) is gaining popularity in the bioimaging and diagnostic fields. This approach uses surface plasmon resonance (SPR) to enhance sensitivity while inheriting crucial Raman fingerprint information (Kleinman et al., 2013). Au NPs are considered excellent for *in vivo* imaging applications because they are inert, biocompatible, and their localized surface plasmon resonances (LSPRs) may be adjusted toward the near-infrared regions (NIR) window - an optical window (700–950 nm) with low tissue absorption and autofluorescence (Smith et al., 2009). Recently, Au nanocages (Au NCs) have garnered considerable attention for their potential use in constructing theranostics nanoplatforms with adjustable size and shape and increased Raman active chemical content (Au et al., 2010; Pang et al., 2016; Wen et al., 2019) (Figure 2). Due to the self-aggregation property of unmodified Au NCs, however, the colloidal stability in the tumor microenvironment is decreased, lowering the repeatability of detection (Zeng et al., 2013). In this context, Farahavar et al. developed immuno-liposomal layer-coated p-Au NCs by conjugating anti-MUC18 scFv to liposomal layer coated p-Au NCs in order to build a theranostics system for selective SERS imaging and thermal ablation of MUC18-expressing melanoma malignant cells (A375). The findings suggested that the modified SERS nanoprobe was capable of actively identifying and diagnosing malignant melanoma cells expressing MUC18 (Farahavar et al., 2021).

### 3.3 Functionalized Polymeric Nanoparticles

**FIGURE 3:**
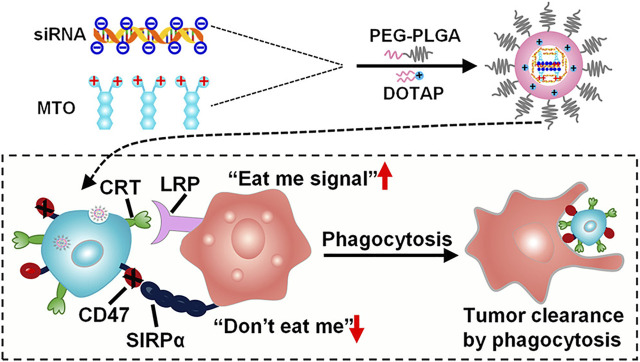
The diagram of PLGA-based nanoparticles for the simultaneous delivery of siRNA and mitoxantrone hydrochloride (MTO·2HCl) (Zhang Y. et al., 2021)

The goal of developing polymeric nanoparticles was to reduce the loss and early degradation of the medicine contained inside them, which normally occurs after chemical and/or enzymatic deactivation. They’ve shown the capacity to improve medication bioavailability, lessen unpleasant side effects, and raise the proportion of drug stored in a specific part of the body (Narayanaswamy and Torchilin, 2021). Because the majority of melanoma anticancer medicines are lipophilic, their antitumor activity is restricted owing to their adverse pharmacokinetic and pharmacodynamic characteristics (Zheng et al., 2009). The use of amphiphilic polymers (which have both hydrophobic and hydrophilic sections) in anticancer medication formulations has effectively altered the release profile of free medicines (Zhang Y. et al., 2021) (Figure 3). Various forms of polymer nanoparticles, such as nanospheres and nanocapsules, polymer micelles, polymers, dendrimer-based micelles, and polymer drug conjugates, may be manufactured depending on the characteristics of the polymer and their uses (Pucek et al., 2020; Toro et al., 2021). Alves Batista et al. have suggested the usage of NPs made of poly (methyl methacrylate) (PMMA) (Alves Batista et al., 2020), with the ability to incorporate α-terpineol, a monoterpenoid known in the literature for exerting beneficial effects against leukemic cell lines (Nogueira et al., 2014). When α-terpineol is integrated into PMMA/-terpineol NPs and evaluated in melanoma-derived tumor cell lines, the findings imply that it boosts anticancer activity. Furthermore, no toxicity was seen in normal cells (human macrophages and MRC-5 human fibroblasts), suggesting that this formulation might be highly effective in reducing the adverse effects caused by many antineoplastic medications when given in their free forms.

Given vitamin D and its analogs’ antiproliferative and differentiation-promoting properties, it appeared prudent to investigate their effectiveness as anticancer agents and their potential for favorable interactions with other anti-melanoma agents or treatment methods (Pettijohn et al., 2014). Scopel et al. created Lipid-polymer hybrid NPs made of a poly (lactic-co-glycolic) acid (PLGA) core and a lipid combination-hydrogenated soy phosphatidylcholine (HSPC), CHOL, and DSPE-PEG_2000_-as a shell. Vitamin D3 functionalized lipid-polymer hybrid nanoparticles were also created to target the vitamin D receptor (VDR) and promote cell internalization, in which Vitamin D3 was covalently linked to DSPE-PEG_2000_ (Scopel et al., 2022). HNP-VD was localized in the perinuclear area of B16 melanoma cells, presumably owing to the presence of the vitamin D ligand that targets nuclear receptor VDR. These findings indicate that HNP-VD is an excellent option for the establishment of tailored melanoma treatment regimens, as well as the delivery of encapsulated therapeutic molecules to other cells expressing nuclear vitamin D receptors.

### 3.4 Functionalized Carbon Nanotubes

Carbon nanotubes (CNTs) are molecular tubes made up of one or more graphene helical sheets (a single layer of carbon atoms). Single-walled carbon nanotubes (SWCNTs) and multi-walled carbon nanotubes (MWCNTs) are the two types (Beiu et al., 2020). Because of their biocompatibility and ability to carry vast cargos of medicines and biomolecules, CNTs have become more essential in recent years. (Degim et al., 2010).

**FIGURE 4:**
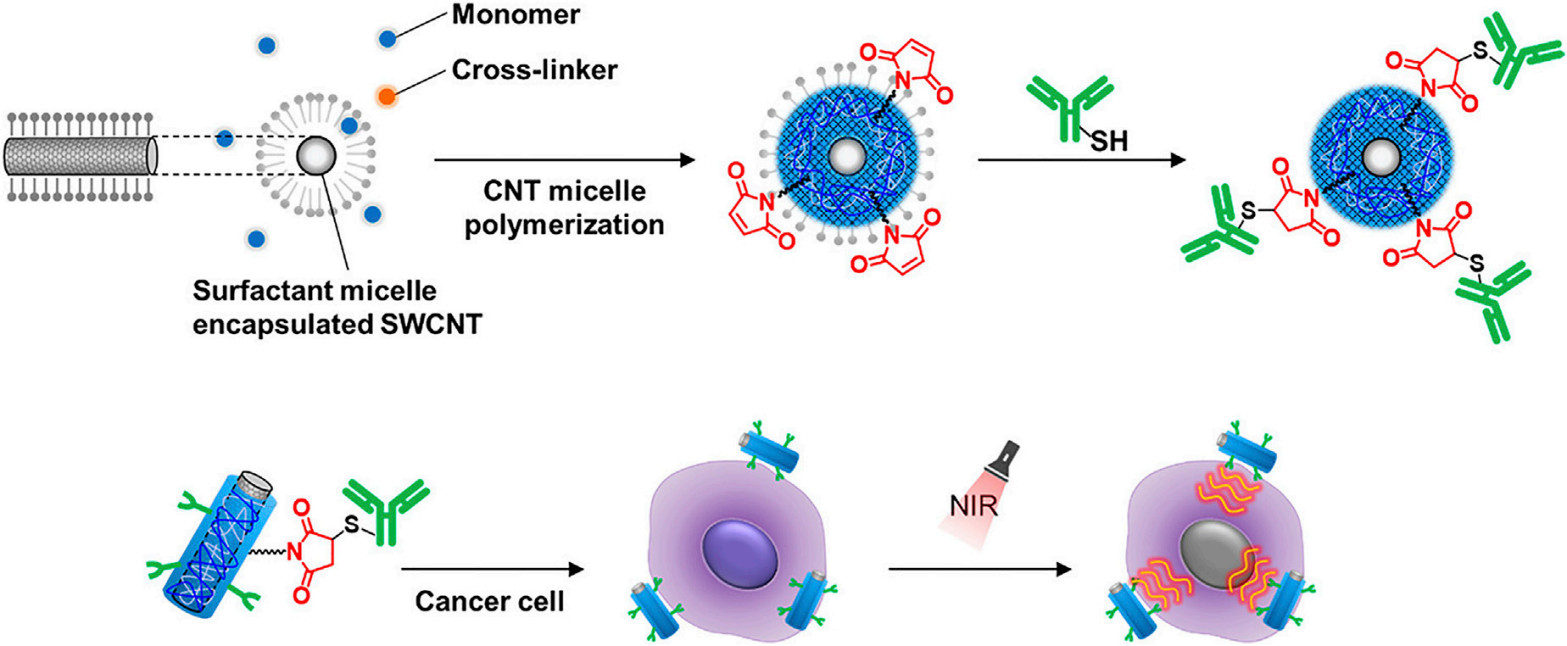
Illustration of the process used for the modification of single-walled carbon nanotubes (SWCNTs) with antibodies using the ene-thiol reaction and the use of the modified SWCNTs in the PTT-mediated killing of cancer cells (Nagai et al., 2021).

SWCNTs are promising candidates for NIR photothermal agents due to their excellent absorption and photothermal conversion efficiency (Jeng et al., 2006). Modifications of SWCNTs are necessary for the creation of the SWCNT-based PTT materials in order to provide a stable dispersion for biocompatibility and to target the desired tumor while preserving their NIR absorbance (Li et al., 2019; Zhao et al., 2021). However, under physiological conditions, noncovalent or covalent functionalized SWCNTs may be quickly reversed or impair the NIR absorption capabilities (Zhou et al., 2008). To overcome these limitations, Nagai et al. have designed an antibody-conjugated gel-coated SWCNTs exhibiting both stable properties and a high NIR absorption signal (Nagai et al., 2021) (Figure 4). Using the PTT approach, the researchers proved that the antibody-conjugated gel-coated SWCNT was effective in targeting cancer cells and destroying them. This innovative approach for conjugating antibodies to SWCNTs will serve as the foundation for the development of an SWCNT-based platform for the development of NIR photothermal agents in the near-infrared.

## 4 Conclusions and Outlooks

Nanotechnology has expanded the medical business by opening up new opportunities for treating a broad variety of ailments and illnesses. Emerging nanotechnological approaches are crucial in exhibiting robust anti-carcinogenic processes, with advantages such as tumor-specific medicine administration, increased treatment efficacy, fewer adverse event rates, and reduced tumor invasional dispersion. In comparison to standard therapy, careful selection of suitable nanocarriers for loading appropriate chemotherapeutic medicines has shown promising results in terms of dosage reduction. This area has the ability to detect proliferative episodes and improve the survival rate of skin cancer patients while also reducing the burden on medical facilities.
